# Minimally Invasive Microendoscopic Resection of the Transverse Process for Treatment of Low Back Pain with Bertolotti's Syndrome

**DOI:** 10.1155/2014/613971

**Published:** 2014-06-19

**Authors:** Yoichiro Takata, Toshinori Sakai, Kosaku Higashino, Yuichiro Goda, Kazuaki Mineta, Kosuke Sugiura, Koichi Sairyo

**Affiliations:** Department of Orthopedic Surgery, The University of Tokushima, 3-18-15 Kuramoto-cho, Tokushima 770-8503, Japan

## Abstract

Bertolotti's syndrome is characterized by anomalous enlargement of the transverse process of the most caudal lumbar segment, causing chronic and persistent low back pain or sciatica. We describe the case of a 45-year-old woman who presented with left sciatic pain and low back pain due to a recurrent lumbar disc herniation at L4-5 with Bertolotti's syndrome. Selective L5 nerve root block and local injection of lidocaine into the articulation between the transverse process and sacral ala temporarily relieved the left sciatic pain and low back pain, respectively. To confirm the effect of local injection on low back pain, we gave a second local injection, which once again relieved the low back pain. Microendoscopic resection of the pseudoarticulation region and discectomy successfully relieved all symptoms. This report illustrates the effectiveness of minimally invasive resection of the transverse process for the treatment of low back pain with Bertolotti's syndrome.

## 1. Introduction

Bertolotti's syndrome, first reported in 1917, is characterized by anomalous enlargement of the transverse process of the most caudal lumbar vertebrae with a lumbosacral transitional vertebra (LSTV) described as “sacral assimilation of the lumbar vertebrae.” The enlarged transverse process sometimes fuses or articulates unilaterally or bilaterally with the sacral ala or iliac crest. The cause of low back pain with Bertolotti's syndrome remains controversial, and various treatments such as local injection of anesthetic and/or steroid, radiofrequency coagulation, surgical resection, and spinal fusion have been reported [1–8]. We present a case of recurrent herniated nucleus pulposus (HNP) at L4-5 with Bertolotti's syndrome treated by microendoscopic discectomy and resection of the transverse process.

## 2. Case Presentation

**Figure 1:**
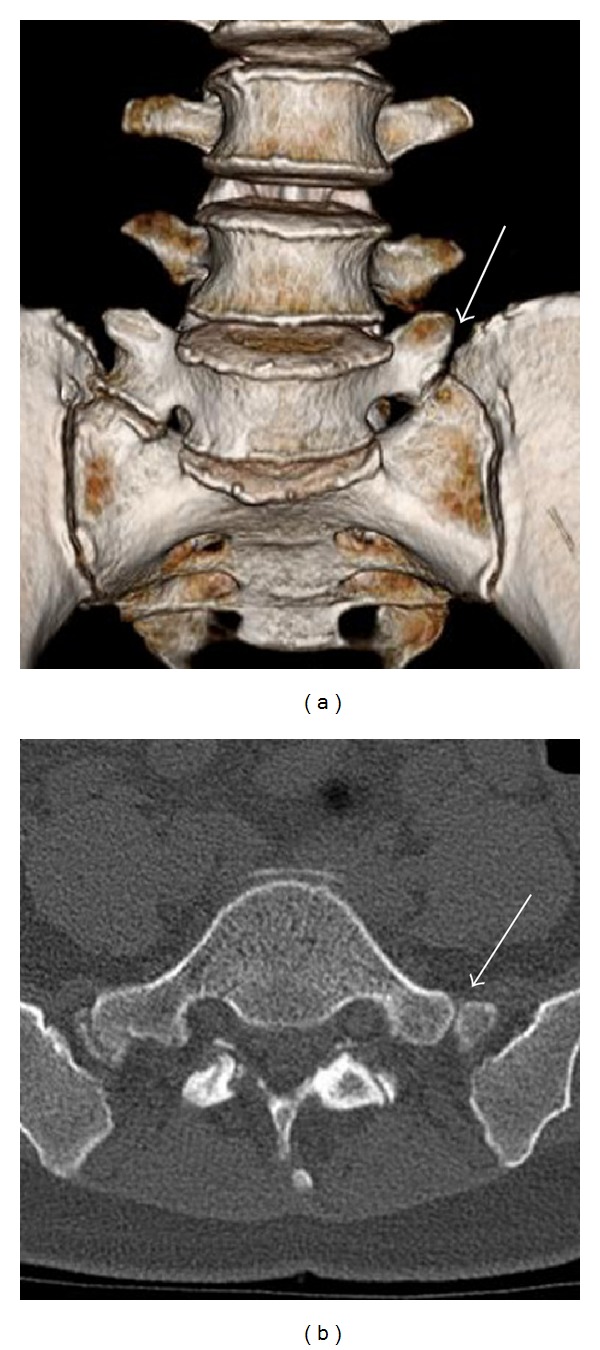
Three-dimensional reconstructed image (a) and axial image (b) showing bilateral articulation (arrow).

**Figure 2:**
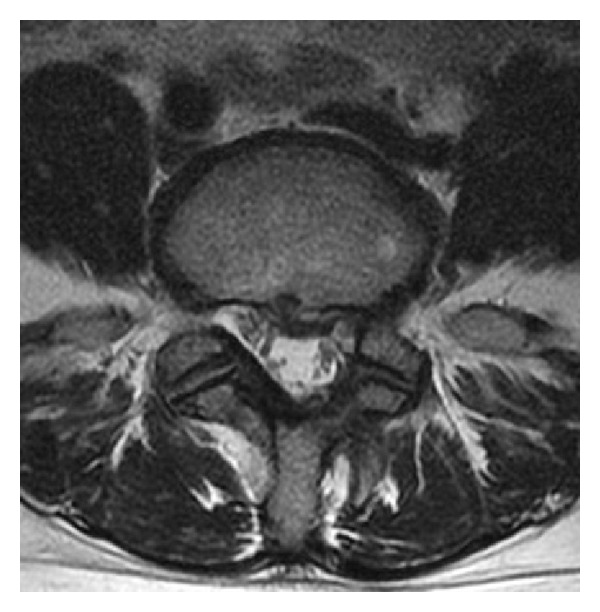
Preoperative magnetic resonance image showing a small recurrent herniated nucleus pulposus at L4-5 compressing the left L5 nerve.

**Figure 3:**
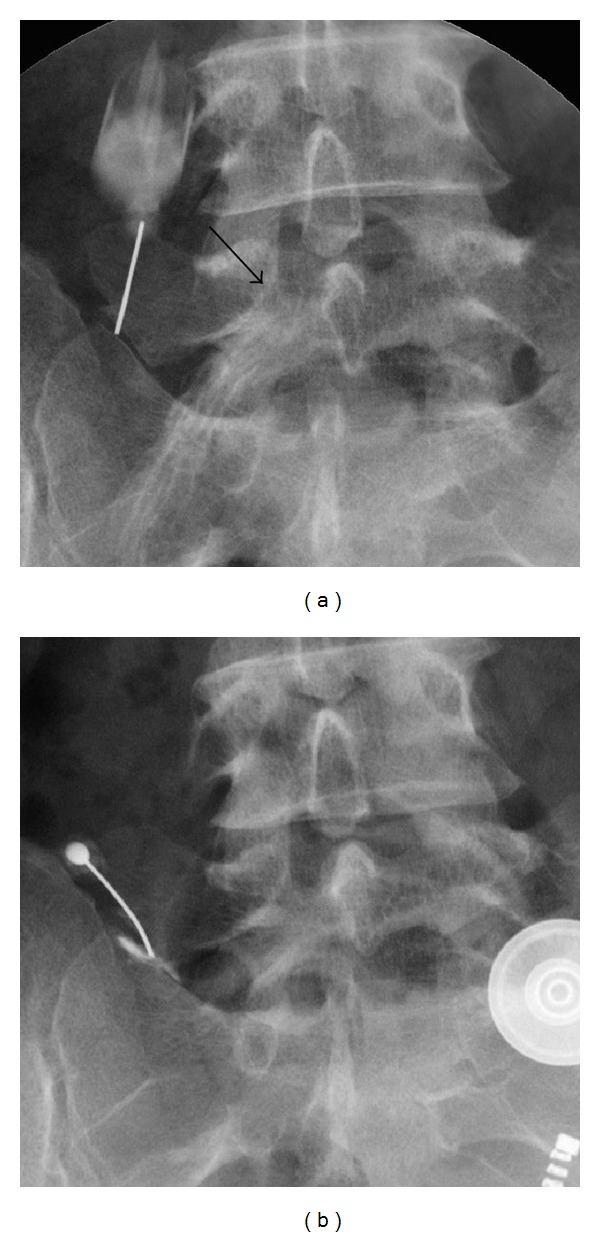
Selective radiculograph of the left L5 nerve root and local injection into the gap between the left transverse process and sacral ala (a) showing impingement of the left L5 nerve root at the lateral recess (arrow) and no impingement in the articulation region. The effectiveness of local injection into the gap between the left transverse process and sacral ala was reconfirmed (b).

A 45-year-old woman presented with a 6-month history of left sciatic pain. Physical examination revealed normal reflex, sensation, and muscle strength in both lower limbs, and the straight leg raise test was positive with radiating pain in the left leg at 45 degrees. Magnetic resonance imaging (MRI) revealed an HNP on the left side at L4-5. Leg pain was relieved postoperatively by microendoscopic discectomy. Three months later while lifting furniture, her leg pain recurred and left low back pain appeared. Tenderness over the left transverse process was apparent, and plain radiography showed enlarged transverse processes at L5. Dynamic radiography showed no instability or slippage at L4-5. Computed tomography (CT) revealed enlarged transverse processes of the L5 vertebra, which articulated bilaterally with the sacral ala, mainly on the right side (Figure 1). MRI showed recurrence of the small HNP on the left side at L4-5 compressing the left L5 nerve root (Figure 2). Selective nerve root block for the left L5 nerve provided temporary relief of leg pain, and local injection of lidocaine into the gap between the left transverse process and sacral ala achieved temporary relief of low back pain from 80/100 to 40/100 on the visual analogue scale (VAS) (Figure 3(a)). Radiculography of the left L5 nerve root showed impingement at the lateral recess and no impingement around the articulation. To confirm the effectiveness of local injection, we conducted local injection alone, which again temporarily relieved the low back pain (Figure 3(b)).

**Figure 4:**
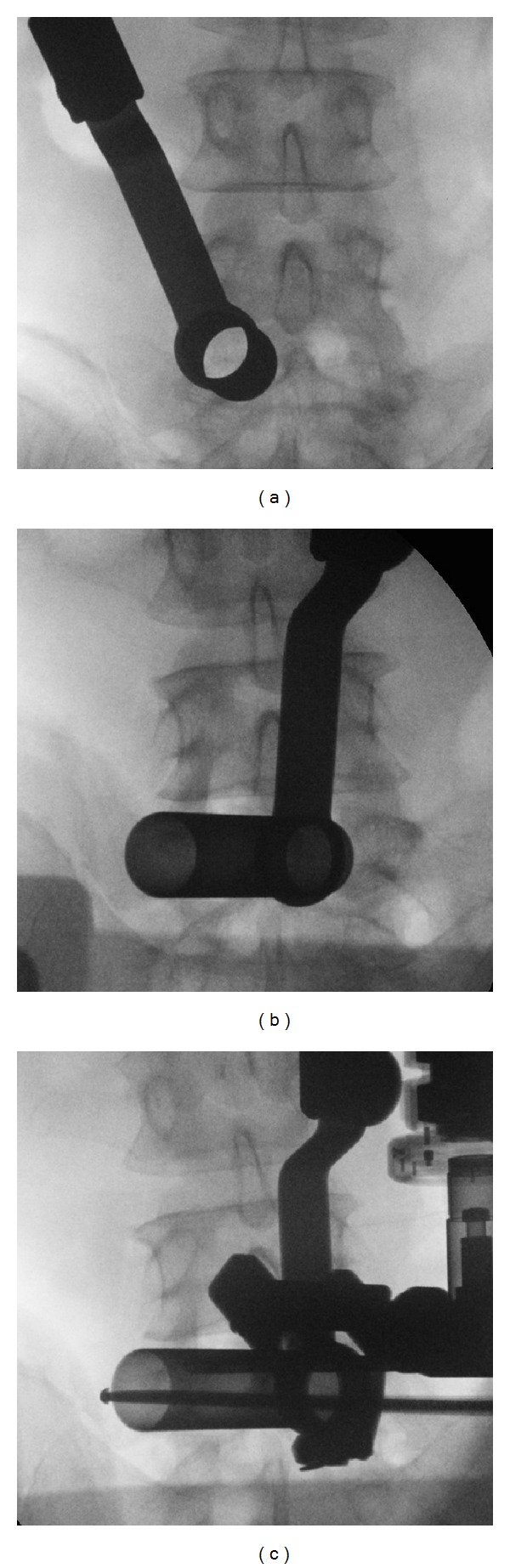
Intraoperative fluoroscopic images showing installation of the tubular retractor over the interlaminar space at left L4-5 for discectomy (a) and over the articulation for resection of the transverse process (b). The transverse process and sacral ala were resected with a high-speed burr (c).

**Figure 5:**
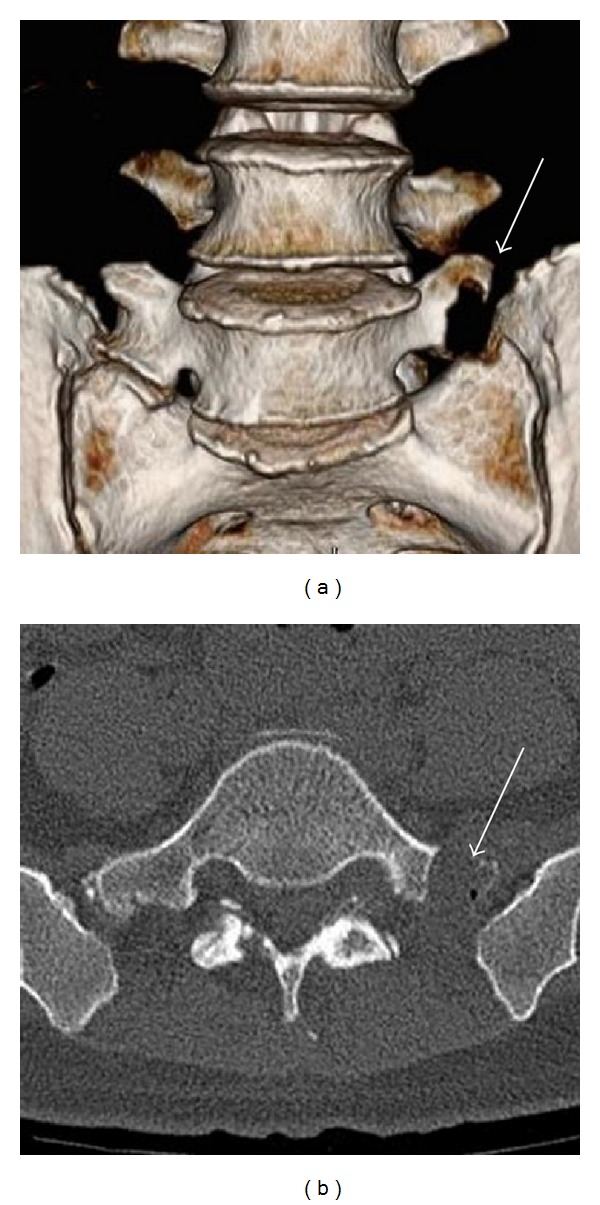
Postoperative three-dimensional reconstructed image (a) and axial image (b) showing successful resection of the articulation (arrow).

On the basis of these findings, we concluded that the leg pain was derived from the recurrent HNP and that the low back pain was derived from the articulation between the transverse process and the sacral ala, so called Bertolotti's syndrome. We then conducted discectomy at L4-5 and resection of the left L5 transverse process using the ESD II system (Japan Medical Dynamic Marketing Inc., Japan). A 2 cm skin incision was made 2 cm to the left of the midline, tissue layers were sequentially dilated, and a tubular retractor was introduced over the interlaminar space of the left L4-5 (Figure 4(a)). Partial laminectomies of L4 and L5 and medial facetectomy of L4-5 were made. The scar tissue was carefully retracted, and the left L5 nerve root was decompressed by removing the HNP. To approach the transverse process and the sacral ala, the tubular retractor was tilted laterally to overcome the L4-5 facet joint (Figure 4(b)). The caudal part of the transverse process and the articulated lesion of the sacral ala were removed with a high-speed burr under fluoroscopic guidance (Figure 4(c)). Postoperative CT images showed successful resection of the articulation of the transverse process and sacral ala (Figure 5), and her low back pain and sciatic pain improved from 80/100 to 29/100 and from 80/100 to 10/100, respectively on the VAS.

## 3. Discussion

Castellvi et al. analyzed a series of 200 patients with positive myelographic findings of lumbar disc herniation and discovered lumbosacral anomalies in 60 cases [9]. Castellvi et al. proposed a classification of four groups based on the morphological characteristics of such abnormal vertebrae. Bertolotti et al. first described the association between low back pain and transitional vertebra in 1917. Although the presence of articulation between the transverse process and the sacral ala has been suggested to contribute to low back pain [9–12], results have been conflicting [13].

The prevalence of Bertolotti's syndrome was reported to be 4–8% in adults with low back pain [14, 15]. Quinlan et al. reported that 4.6% of patients with low back pain had Bertolotti's syndrome and that the incidence was especially higher in younger patients [11].

Although the exact cause of this syndrome is unclear, the following etiologies have been put forward: (1) articulation of the transverse process and sacral ala [1, 4, 5, 11, 16]; (2) degeneration of the intervertebral disc at adjacent levels [10]; (3) facet joint contralateral to a unilateral fused or articulating LSTV [7]; and (4) extraforaminal stenosis secondary to the presence of a hypertrophied transverse process [8, 12, 17].

Conservative treatment of Bertolotti's syndrome, such as with nonsteroidal anti-inflammatory drugs or local injection into the articulation, is typically the first choice [1, 16]. Marks and Thulbourne reported that steroid injection into the articulation provided temporary relief, which may last up to two years [1]. However, if conservative treatment is ineffective, surgical treatment should be considered.

Two types of surgical treatment for low back pain with Bertolotti's syndrome have been reported. In particular, several authors have reported relatively favorable outcomes following resection of the articulation between the transverse process and sacral ala [3–6, 8, 18, 19]. The outcome of posterolateral fusion was reported by Santavirta et al., who showed no significant differences compared with resection of the articulation [3].

In our case, the patient had bilaterally enlarged transverse processes of the L5 vertebra mainly on the right side. Although her low back pain was localized to the left side, the local anesthetic injection to the articulation of left L5 transverse process and sacrum achieved half pain relief. That suggests the Bertolotti's syndrome of the right side is also associated with low back pain. And resection of the left transverse process might bring the overload to the right articulation biomechanically and lead to recurred low back pain in the future.

In summary, we diagnosed a case of low back pain due to Bertolotti's syndrome and sciatica due to a recurrent HNP by local injection and selective nerve root block, respectively. Increased biomechanical loading on the caudal disc above the LSTV might have led to HNP recurrence in this case. To minimize surgical invasiveness, we conducted microendoscopic resection of the transverse process, followed by microendoscopic discectomy via the same skin incision.

## 4. Conclusion

Management of patients with Bertolotti's syndrome should be carefully considered. Adequate interventions may be required to elucidate the pain source. In cases of insufficient pain relief, surgical treatment such as resection of the transverse process or spinal fusion should be considered. The present case is a successful example of minimally invasive resection of the transverse process.
